# Investigation of spin stiffness in spin-depolarized states of two-dimensional electron systems with time-resolved Kerr rotation

**DOI:** 10.1038/s41598-020-58658-4

**Published:** 2020-02-10

**Authors:** A. V. Larionov, E. Stepanets-Khussein, L. V. Kulik, V. Umansky, I. V. Kukushkin

**Affiliations:** 1 Institute of Solid State Physics, Russian Academy of Sciences, 142432 Chernogolovka, Russia; 2 National Research University Higher School of Economics, 101000 Moscow, Russia; 3 Braun Center for Submicron Research, Weizmann Institute of Science, Rehovot, 76100 Israel

**Keywords:** Spintronics, Quantum Hall

## Abstract

An experimental technique based on time-resolved Kerr rotation allows a comparison of the spin stiffnesses of different spin-polarized and depolarized states in a two-dimensional electron system. With this technique, a new spin-correlated phase that has no known analogues was discovered. The new spin-depolarized phase is characterized by high spin stiffness equal to that of a spin-polarized quantum Hall ferromagnet.

## Introduction

Recently, much attention has been devoted to technological applications based on the manipulation of spin degree of freedom, which has particularly boosted the development of magnonics, the use of spin waves (magnons) for signal transfer^1–3^. More exotic applications entail the involvement of skyrmions, which are spin vortex textures that are topological charge carriers, in spin dynamics. Experimental work on the manipulation of skyrmions and measurement of their mass and characteristic drift length in crossed magnetic and electric fields has been reported^4^.

Most of the aforementioned experimental achievements in manipulation of spins and their textures have been for three-dimensional magnetic systems. In contrast, for a two-dimensional electron system (2DES) in a magnetic field, which has given rise to spin-texture physics, the experimental progress is not so impressive. This is largely due to the absence of reliable experimental techniques revealing the local properties of 2DES spin subsystems, such as Lorentz transmission electron microscopy^5^. Setting aside the transport techniques that are not very sensitive to spin ordering, the key method for the characterization of a spin subsystem involves the measurement of the 2DES magnetization as a function of temperature and filling factor and comparison of the experimental data with those obtained from existing theories^6,7^. A quantum Hall ferromagnet (QHF) with a filling factor of *ν* = 1 is chosen as a reference point of a fully spin-polarized state with maximum achievable spin stiffness. However, for a spin depolarized 2DES, little experimental opportunities for revealing the physical reason for this depolarization exist.

A breakthrough in description of spin-depolarized states was achieved when experimentalists focused on the investigation of the spin relaxation processes in a 2DES determined by the local properties of the spin subsystem. The pioneering works on nuclear spin relaxation via a contact interaction with the spins of an electron system led to the concept of skyrmions and skyrmion crystal^8,9,10^. Although the theory of skyrmion crystals was developed for two-dimensional electron systems, the actual formation of a skyrmion crystal lattice was found in three-dimensional MnSi ferromagnetic films and similar compounds with Dzyaloshinskii–Moriya interaction^5,11,12^. Thus far, there has been no compelling evidence on the existence of a skyrmion crystal in a 2DES formed by either lack or excess of electron density in a quantum Hall ferromagnet. Moreover, observations of spin excitation spectra using the inelastic light scattering technique enabled the assumption that the ground state of a quantum Hall ferromagnet with charge defects is spin-texture (ST) liquid rather than a skyrmion crystal^13,14^. A 2DES exhibits new gapped spin excitation branches related to the spin precession around spin textures (ST). The Berry phase acquired by an electron spin while circling around the spin texture transforms into a fictitious magnetic field proportional to the spin texture density rather than the external magnetic field. The behavior of spin excitations is similar to that of cyclotron excitations in this fictitious magnetic field. Accordingly, the effective “spin mass” participating in “cyclotron transitions” is determined by the magnetization of 2DES and by the spin stiffness^15^. Thus, to advance the understanding of profound states as spin texture liquids, skyrmion crystal, and other spin depolarized states of 2DES, such as those predicted by Frank Wilczek and others,^16^ an experimental technique to directly address spin stiffness is required.

A significant advancement in the investigation of spin stiffness was achieved with the time-resolved Kerr rotation^17,18^. However, the development of a theory describing the physics of time-resolved Kerr rotation has taken some time, even for understanding the easiest 2DES state quantum Hall ferromagnet^19,20^. It is understood now that a pump laser pulse reduces the total spin of the 2DES from its equilibrium direction, whereas a delayed probe pulse can be used to test the dynamics of spin precession around the direction of the external magnetic field. The spin precession dephasing time is determined by spatial fluctuations of the electron *g*-factor. In the absence of electron-electron interaction, the collective 2DES precession splits into individual precessions of separate spins with local Zeeman frequencies and rapidly dephases. Conversely, the exchange electron-electron interaction (spin stiffness) forces the electron spins to be co-directional despite the local fluctuations of the *g*-factor, thus preventing the dephasing of the collective spin precession. Thus, spin stiffness and the spin precession dephasing time are closely related. In our work, we extend the study of spin stiffness to spin-depolarized electronic phases through spin precession dephasing time by means of time-resolved Kerr rotation. We discover that even when the 2DES is strongly depolarized (i.e., the spin polarization is only slightly different from one-particle polarization) the spin stiffness of the 2DES may remain so high that it suggests the existence of a spin-depolarized, but at the same time, a highly spin-correlated, new electronic phase that has no known analogs in the spin physics of 2DES in magnetic field.

We investigated GaAs/AlGaAs heterostructures with a single quantum well of 18 nm width containing highly mobile 2D electron gas [transport mobility *μ*_*e*_ ≃ 5 ⋅ 10^6^ cm^2^/(V ⋅ s)] with dark electron concentration *n*_*s*_ ≃ 0.6 ⋅ 10^11^ cm^−2^ (the electron effective mass in GaAs is 0.067*m*_*e*_). Kerr signal measurements were made in a spectrally degenerate mode with coinciding wavelengths of the pumping and probing laser beams. The photoexcitation source was a picosecond titanium-sapphire laser with a tunable excitation wavelength (around 810 nm). The laser wavelength was chosen to excite resonantly two allowed electron transitions from two spin states of the zero Landau level of the heavy holes states in the valence band of the quantum well to the empty spin states of the zero Landau level in the conductance band of the quantum well. The laser pulse repetition rate was 82 MHz. The spectral width of 1 meV was chosen to overlap two optical transitions involving both spin states in the zero Landau level of electrons. A circularly polarized pumping pulse was used to create a spin orientation of 2D electrons, which was measured by the Kerr rotation angle of a linearly polarized laser beam reflected from the sample with the aid of a special balance photodetector. The average pumping power was 0.6 mW, with a laser spot size of the order of 30 *μ*m. The Kerr signal measurement involved double synchronous detection that enabled the effective suppression of the parasitic scattered laser light from the sample surface. This was achieved by the additional amplitude modulation of the probing beam with a certain modulation frequency, at which the Kerr-rotation signal was recorded. The experiment was performed in a magnetic field using a cryostat with a superconducting solenoid and an temperature insert for ^3^He isotope condensation. The temperature insert design allowed a gradual temperature change from 0.6 K to 15 K. The sample holder had an optical window to introduce laser photoexcitation into the cryostat and collect the Kerr rotational signal. The sample under study was mounted in the holder so that the perpendicular to the quantum well plane was oriented at 45° with respect to the magnetic field axis. The presence of a transverse component of the magnetic field resulted in a coherent Larmor precession of 2D electrons.

Following is a short introduction to the physics of Kerr rotation in a magnetized 2DES in accordance with theory developed in ref. ^20^. The Kerr rotated state is nonstationary even in the absence of any dissipation processes: in the leading approximation, the spin evolution is a precession motion described by the equation of motion ∂**S**/∂*t* = −*gμ*_B_**S** × **B**, which, for spin components, is reduced to ∂*S*_*z*_/∂*t* = 0 and ∂**S** _⊥_∕∂*t* = − *gμ*_B_**S** _⊥_ × **B**. The transverse component **S**_⊥_ = (*S*_*x*_, *S*_*y*_) appears when microscopic excitations meet changes in spin quantum numbers for which |*δS*| < |*δS*_*z*_|. These states are generated by operator ${ {\hat{S}} }_{-}={ {\hat{S}} }_{x}-i{ {\hat{S}} }_{y}$, preserving the total spin of the system, but changing the *S*_*z*_ component by unity: *S*_*z*_ → *S*_*z*_ − 1. For instance, for a quantum Hall ferromagnet in which the ground state has the form $\left|0\right\rangle =\left|{\overbrace{\uparrow \uparrow \uparrow \cdots \uparrow }}^{{{\mathcal{N}}}_{\varphi }}\right\rangle$ (${{\mathcal{N}}}_{\varphi }$ is the number of states with equal spins in the Landau level), the *n*-fold action of operator ${ {\hat{S}} }_{-}$ creates a stationary eigenstate $\left|\,n\right\rangle ={({ {\hat{S}} }_{-})}^{n}\left|\,0\right\rangle$ with the same orbital wave function and total spin value $S={{\mathcal{N}}}_{\varphi }/2$ as those of $\left|0\right\rangle$ yet ${S}_{z}={{\mathcal{N}}}_{\varphi }/2-n$. In this case, the energy is equal to *E*_0_ + *ϵ*_Z_*n*, where *E*_0_ is the ground state energy and *ϵ*_Z_ is the Zeeman energy. Moreover, any combination of such states,

1 $$\sum _{n}{C}_{n}\left|\,n\right\rangle ,$$

specified by a set of coefficients {*C*_*n*_}, is also a state with an orbital wave function and total spin *S* being the same as that in the ground state. If the number of terms in this sum is more than one, it no longer corresponds to any eigenstate of the Hamiltonian even by neglecting spin non-preserving interactions. In general, for such superpositions of different eigenstates, there is no ${ {\hat{z}} }^{^{\prime} }$-direction in the spin space where its spin projection ${S}_{{z}^{^{\prime} }}$ would be a quantum eigenvalue, i.e., where the spin in that direction has a definite value (in quantum mechanics, they are called states with partial spin polarization^21^). The expression (1) fits well with the state appearing in the system under the laser pulse and can be considered as the initial condition for solving the nonstationary Schrödinger equation $i\partial \left|\,N,t\right\rangle /\partial t= {\hat{H}} \left|\,N,t\right\rangle$ (hereafter, we assume *ℏ* = 1). Accordingly, by neglecting all the terms of the Hamiltonian not commuting with operators ${ {\hat{S}} }_{z}$ and ${\widehat{{\bf{S}}}}^{2}$, we obtain a solution in the form

2 $$\left|\,N,t\right\rangle ={e}^{-i{E}_{0}t}\,\mathop{\sum }\limits_{n=0}^{N}{C}_{n}{e}^{-in{\epsilon }_{{\rm{Z}}}t}\left|\,n\right\rangle .$$

This expression provides the most general microscopic description of the Goldstone mode of the quantum Hall ferromagnet. By calculating the quantum mechanical mean value of operator ${ {\hat{S}} }_{+}={ {\hat{S}} }_{x}+i{ {\hat{S}} }_{y}$ in state (2), we obtain the value of the transverse spin at time *t*

3 $$\begin{array}{lll}{S}_{\perp }(t) & = & \left\langle t,N\right|\,{ {\hat{S}} }_{+}\left|\,N,t\right\rangle \\  & = & {e}^{-i{\epsilon }_{z}t}\mathop{\sum }\limits_{n=0}^{N-1}{C}_{n}^{\ast }{C}_{n+1}\left\langle n+1| n+1\right\rangle ,\end{array}$$

which corresponds to precession with Zeeman frequency. The inclination angle with respect to the ${\hat{z}}$-axis is defined as $\theta =\arcsin (| {S}_{\perp }| /S)\equiv \arcsin (2| {S}_{\perp }| /{{\mathcal{N}}}_{\varphi })$, and the quantum mechanical mean value of component *S*_*z*_ is time independent and equal to

4 $${\bar{S}}_{z}=\langle t,N| {S}_{z}| N,t\rangle =\sum _{n}| {C}_{n}{| }^{2}({\mathcal{N}}/2-n)\langle n| n\rangle .$$

Different microscopic states (2) may formally correspond to the same Goldstone mode. Indeed, the same *θ* value can be realized for different sets of {*C*_*n*_} because at a specified angle for a macroscopically large number *N*, there are only two conditions for coefficients *C*_*n*_: (i) ${\sum }_{n}{C}_{n}^{* }{C}_{n+1}\langle n+1| n+1\rangle =({{\mathcal{N}}}_{\varphi }/2)\sin \theta$, and (ii) normalization condition ∑_*n*_|*C*_*n*_|^2^〈*n*|*n*〉 = 1.

Note that each state $\left|\,n\right\rangle$ can be considered as that of a system with excited *n* “Goldstone excitons”. The Goldstone exciton, representing the result of a single action of the ${ {\hat{S}} }_{-}$ spin operator, changes the spin numbers of the state according to the rule *δS*_*z*_ = −1, *δS* = 0, and in no way affects the translation symmetry of the system, i.e., it is an excitation with a two-dimensional momentum equal to zero (this excitation is created in the 2DES after laser pumping pulse of the Kerr rotation experiment). It means that the excitation wavelength is well above the characteristic scale of spatial fluctuations of two-dimensional electron density related to 2DES inhomogeneity. On the other hand, it is known that the system also has magnon-type excitations, purely electronic spin waves with a non-zero two-dimensional vector **q**, which are associated with a unity change in the *S*_*z*_-component and total spin *S*:  *δS* = *δS*_*z*_ = −1^22,23^. The solution of the nonstationary Schrödinger equation, with due account of spatial fluctuations of the Lande *g*-factor, reveals that transformation of a Goldstone exciton into a spin wave is an elementary process leading to the dissipation of the Goldstone mode^20^. It is clear that the *S*_*z*_ value remains unchanged, but the total spin of the system is reduced by unity (*S* → *S* − 1). Formulated differently, it is a transformation where, instead of an $\left|\,n\right\rangle$ state, a state with *n* − 1 Goldstone excitons and one spin-wave exciton with nonzero momentum **q** appears. If *q* → 0 (which actually means that *ql*_*B*_ ≪ 1), then such a transformation corresponds to an elementary stochastization saving both the Zeeman energy (*S*_*z*_ remains unchanged) and exchange energy of the system. Goldstone excitons are non-interacting with each other and the spin waves. Thus, the process of transformation of a Goldstone exciton into a spin wave is a single-exciton process. When considered in the $\left|n\right\rangle$— state, this process is independent of the number *n*. Thus, in state (2), the evolution, which slows down further owing to the presence of relaxation-determining perturbations, follows the same time law for each summand. At $N\ll {{\mathcal{N}}}_{\varphi }$ this law denotes an exponential dephasing of the Goldstone mode (exponential vanishing of transverse component |*S*_⊥_|) and finally, transformation of the mode into a state where the system spin is directed along the magnetic field. Here the quantum mechanical mean values of *S* and *S*_*z*_ are the same and equal to *S*_*z*_ (see Eq. (4)). The characteristic dephasing time can be calculated using the amplitude and correlation length of spatial fluctuations of the *g*-factor and by the spin stiffness^19,20^. It should be emphasized that to extend the discussed microscopic theory of Kerr rotation aside of filling factor *ν* = 1 (Hall ferromagnet) to other filling factors is a problem of high complexity because of our little understanding of the 2DES ground state at *ν* ≠ 1.

**Figure 1:**
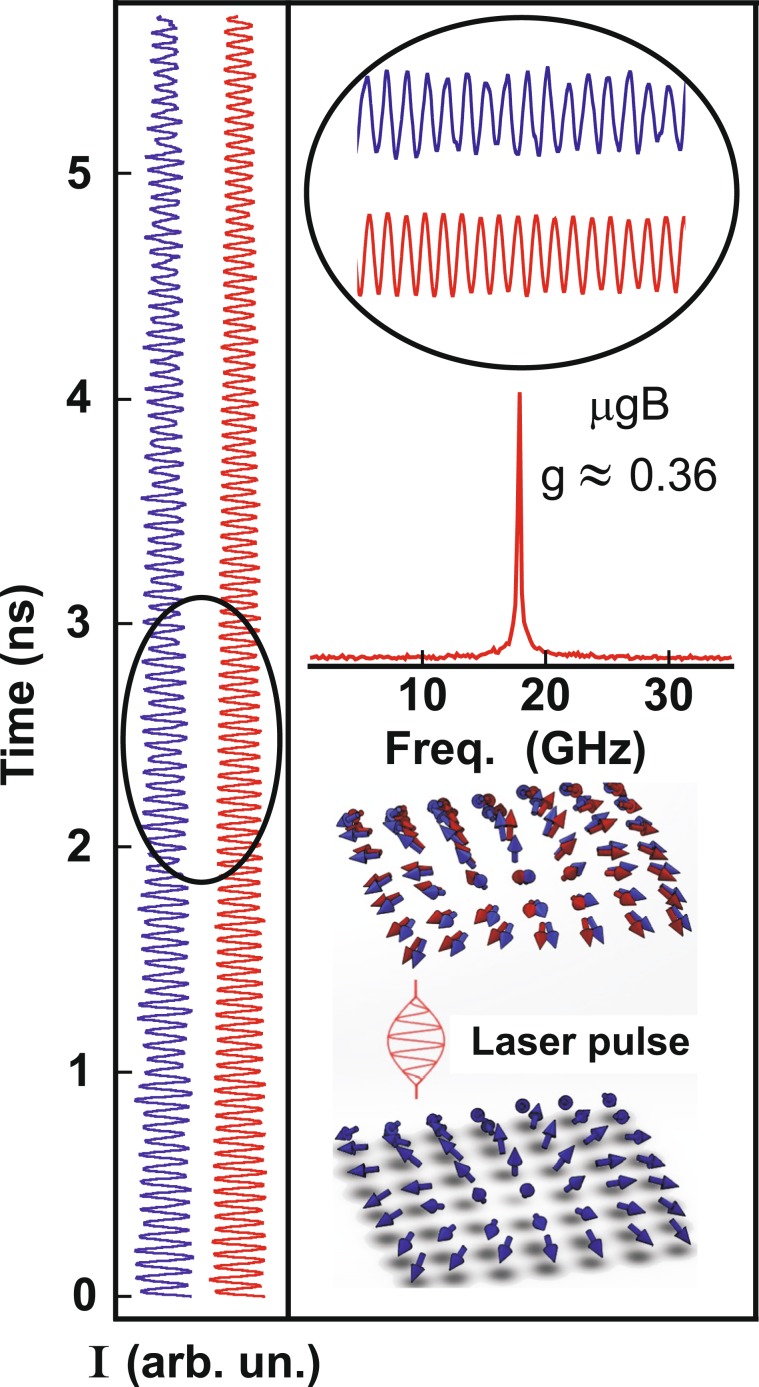
(left) Time dynamics of the Kerr signal involving optical transitions (1/2;3/2) and (1/2; 3/2) (electron-hole transition from the zero Landau level of the conductance band to the zero Landau level of heavy holes of the valence band) measured at *T* = 0.6 K (blue line) and *T* = 4.2 K (red line), *ν* = 1. (right) Fourier transform of the Kerr signal at *T* = 4.2 K. At the bottom: schematic illustration of the effect of the pumping laser pulse on the spin depolarized 2DES (the blue and red arrows show the spin orientation before and after the laser pulse).

**Figure 2:**
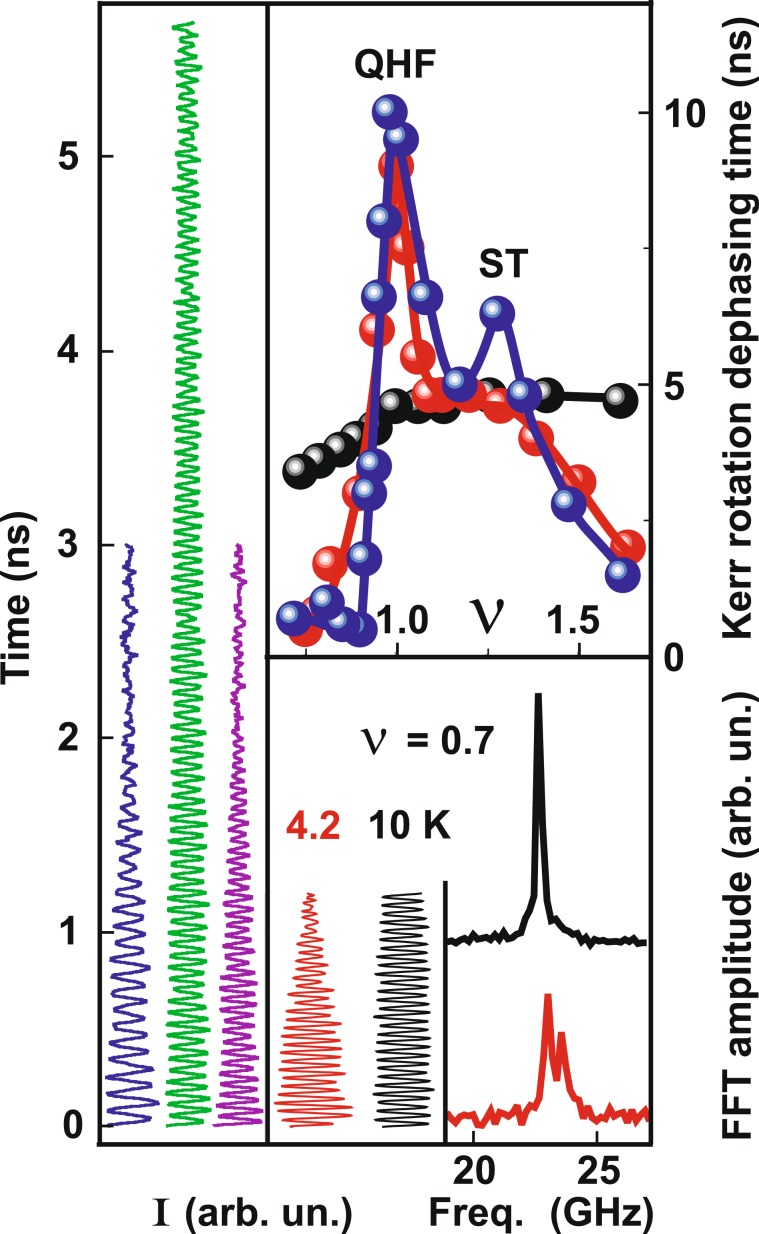
(left) Time dynamics of the Kerr signal measured at *T* = 0.6 K (*ν* = 1 – middle curve, *ν* = 0.9 and *ν* = 1.4 – side curves). (right) Spin dephasing time as a function of the electron filling factor measured at three temperatures *T* = 0.6, 4.2, 10 K (blue, red, and black dots, respectively). At the bottom, the time dynamics of the Kerr signal as well as its Fourier transform at *T* = 4.2 K and 10 K measured in the state with the small spin stiffness at *ν* = 0.7.

The change in spin stiffness of 2DES during transformation from the spin-polarized state (quantum Hall ferromagnet) to spin-depolarized state induced by either adding extra electrons (holes) in the quantum Hall ferromagnet (QHF) or heating the electron system was studied by measuring time-resolved Kerr rotation at different filling factors and electron temperatures. The electron filling factor is determined from the magnetic field dependence of the 2DES luminescence^24^. The spin precession dephasing dynamics is complicated and cannot be described by a simple exponential dependence^20^. Accordingly, the Kerr signal was analyzed by utilizing the difference between the minima and maxima of Larmor oscillations. The spin precession dephasing time was determined as an *e*-fold change of the Larmor precession amplitude. The base line, which is the arithmetic mean between the envelope lines in the upper and lower parts of the oscillating signal, was subtracted from the Kerr curves. The basic results are illustrated in Figs. 1 and 2. In Fig. 1 the Kerr signal is measured at two different temperatures: one corresponds to an almost fully spin-polarized state (*T* = 0.6  K, polarization degree *M*(*T*)/*M*_0_ ≈ 1) and the other corresponds to a depolarized state (*T* = 4.2  K, *M*(*T*)/*M*_0_ ≈ 0.25^6,7^). Surprisingly, in both cases, the spin precession dephasing time is the same. This suggests practically equal spin stiffness for the two cases studied. Figure 2 illustrates the filling factor dependence of spin precession dephasing time in a close neighborhood of the quantum Hall ferromagnet state *ν* = 1. Again, the Kerr signal behaves very similar at two temperatures *T* = 0.6  K and *T* = 4.2  K.

At first sight, unexpected is the increase in the spin precession dephasing time with increasing temperature above *T* = 5*μgB* at non-integer filling factors (Fig. 2, *T* = 10  K). However, we show that it is a natural phenomenon associated with reduced fluctuations of the effective “magnetic field” sensed by the electrons. In addition to the fluctuations of the electron *g*-factor caused by the local compositional disorder of the quantum well barriers as well as fluctuations of the *g*-factor due to changes in the quantum well width, a contribution from the spatial fluctuations of the effective magnetic field induced by nonequilibrium polarization of nuclear spins caused by the laser pulses appears at low temperatures^25–27^. For comparison, Kerr rotation signals were measured at 4.2 K and 10 K at a filling factor of *ν* = 0.7, where the influence of spin correlations on spin precession dephasing can be neglected. At lower temperatures, the fluctuations of the effective magnetic field induced by spatial fluctuations of nuclear spins not only significantly reduce the spin precession dephasing time (by increasing the spectral noise of the 2DES spin precession) but also give rise to an extra peak in the Fourier transform of the Kerr rotation signal corresponding to an extra effective magnetic field of about 0.1 T (Fig. 2). Thus, the spatial fluctuations of the effective magnetic field acting on the electrons from the nuclear spins through the contact interaction are enhanced with decreasing temperature. This leads to faster dephasing of the 2DES spin precession. However, in the quantum Hall ferromagnet, fluctuations of frozen nuclear spins do not significantly affect spin precession because of the high stiffness of the 2DES.

**Figure 3:**
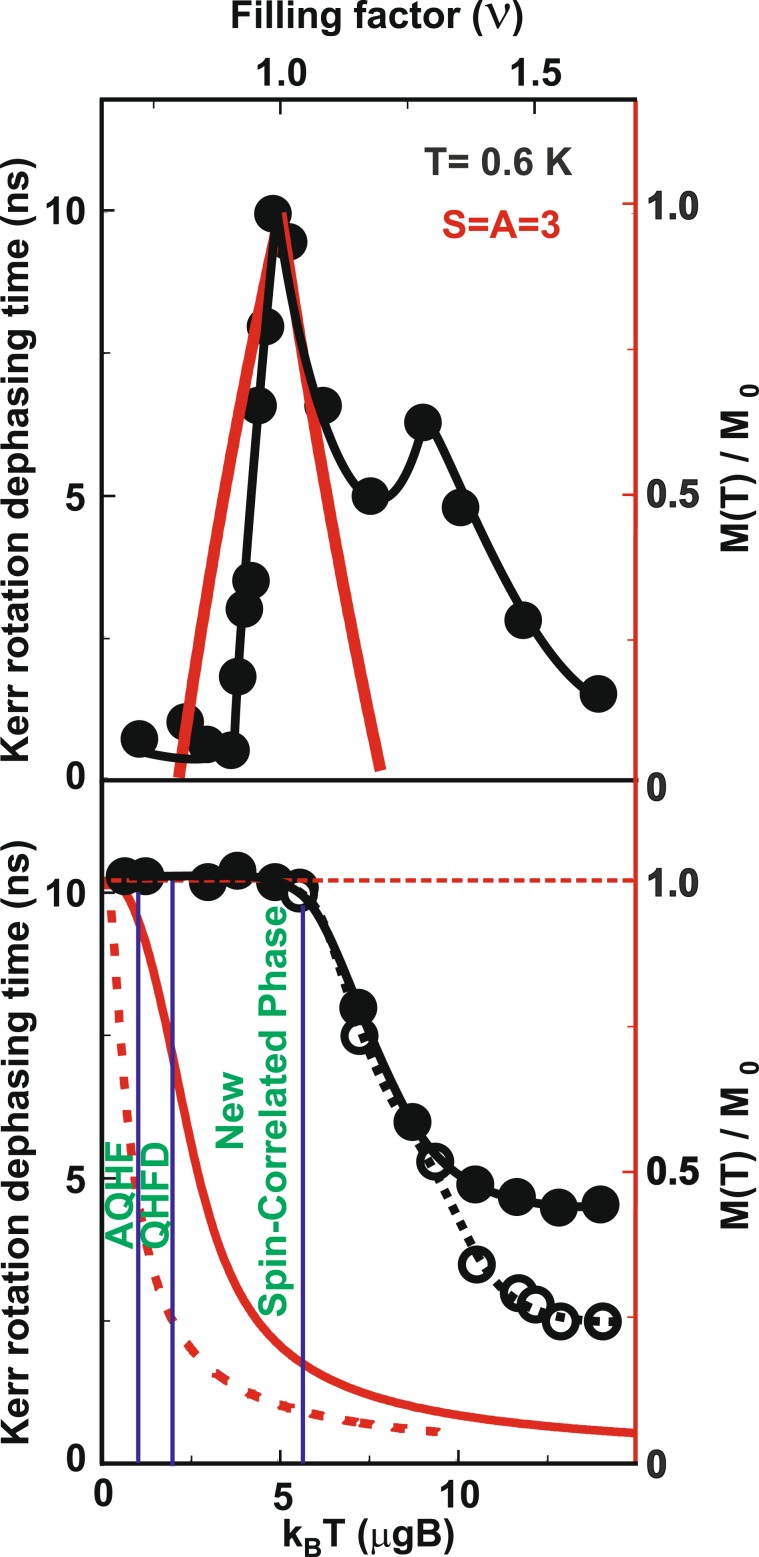
(top) Spin dephasing time as a function of electron filling factor at *T* = 0.6 K (black dots, left axis) and electron system magnetization (red line, right axis) obtained in a skyrmion-based model ^8,9^ with skyrmion spin (*S*) and anti-skyrmion spin (*A*) both equal to 3, which is verified experimentally in ref. ^7^. (bottom) Spin dephasing time versus temperature (expressed in multiples of the Zeeman energy obtained from Fig. 1) at *ν* = 1 (black dots, left axis) and electron system magnetization (red line, right axis) obtained theoretically in ref. ^28^ and experimentally in ref. ^7,29^. The open dots show “fast” temperature scan of the Kerr signal (see text). The dashed line shows single-particle magnetization. The vertical solid lines demarcate different spin phases of 2DES (Activated Quantum Hall Ferromagnet (AQHF), Quantum Hall Ferromagnet Domains identified in ref. ^29^, and a new highly spin-correlated and at the same time, the spin-depolarized electron phase).

The influence of the nonequilibrium subsystem of nuclear spins on the 2DES at high temperatures (above *T* = 5*μgB*) can be enhanced by fast Kerr-signal measurement. The relaxation of nuclear spins to an equilibrium state at helium temperatures is a process that lasts several hours; therefore, the temperature dependence of spin precession dephasing time can be measured in a time scale shorter than that needed for complete nuclear spin relaxation (Fig. 3). The graph of spin precession dephasing time versus temperature in the quantum Hall ferromagnet state shows that at low temperatures (*T* ≤ 5*μgB*), the dephasing time remains the same irrespective of the nuclear spin subsystem state. However, at high temperatures, there is a twofold reduction in the dephasing time in the short-time temperature scan in comparison with the long-time temperature scan, underscoring the importance of nuclear spins for spin precession dephasing in the 2DES with decreased spin stiffness.

It is most remarkable that the spin stiffness at a filling factor of *ν* = 1 survives at such high temperatures when the 2DES is nearly completely depolarized (Fig. 3). This means that during heating, before the quantum Hall ferromagnet turns into a paramagnet, it goes through an extra intermediate phase (new spin-correlated phase) characterized by a high spin stiffness nearly equal to that in the quantum Hall ferromagnet. The same magnetization, as in the new spin-correlated phase, can be obtained at filling factor *ν* = 0.92 by incorporating the holes in the quantum Hall ferromagnet (Fig. 3). Thus, the spin polarization degree of the 2DES at *ν* = 0.92 and T = 0.6 K (spin-texture liquid) is equal to the spin polarization degree of the new spin-correlated phase at *ν* = 1 and T = 6 K; i.e. these two phases are experimentally indistinguishable if one relies only on the polarization degree of the 2DES^6,7^. Besides, such powerful techniques of probing spin states in the 2DES as Raman scattering and electron spin resonance will be of little help for distinguishing these two phases^29^. However, the Kerr rotation technique can do the job. The spin dephasing time for the collective Larmor precession measured in the new spin-correlated phase is 5 times larger than that measured in the spin-texture liquid phase, which suggests much higher spin stiffness for the new spin-correlated phase^19^. This points out that the new spin-correlated phase is not a spin texture liquid forming in the 2DES at low temperatures^13^. It also has no relation with a skyrmion or an anti-skyrmion state, as the spin correlation length for a skyrmion (*S*) or an anti-skyrmion (*A*) in GaAs is about *S* = *A* = 3 or even less owing to the relatively small ratio of the exchange energy to the Zeeman energy (Fig. 3)^30^. The new spin-correlated phase should consist of spin textures with a high spin stiffness, which implies a much larger spin correlation length for a single texture than that for skyrmions and anti-skyrmions (with nearly co-directional spins as in the quantum Hall ferromagnet).

In conclusion, different spin-polarized and depolarized electron states of 2DES in a magnetic field were studied using the time-resolved Kerr rotation technique. A new, strongly spin-correlated phase that consists of spin textures, characterized by high spin stiffness considerably exceeding the spin stiffness of the skyrmion in GaAs quantum wells, was discovered. The transition from the new spin-correlated state to a low spin-stiffness state demonstrates a stepwise temperature dependence (Fig. 3), which may suggest the relation of the new phase with topological spin phases discussed in ref. ^16^. The technique developed in this study for comparison of spin stiffnesses of different spin-polarized and depolarized states through the spin precession dephasing may be employed to study spin arrangements in various spin polarized and depolarized 2DES states with different degree of spin-spin correlations.

## Data Availability

Data are available from the corresponding author upon request.
